# Coronary Artery Perforations: Glasgow Natural History Study of Covered Stent Coronary Interventions (GNOCCI) Study

**DOI:** 10.1161/JAHA.121.024492

**Published:** 2022-09-21

**Authors:** Thomas J. Ford, Carly Adamson, Andrew J. Morrow, Paul Rocchiccioli, Damien Collison, Peter J. McCartney, Aadil Shaukat, Mitchell Lindsay, Richard Good, Stuart Watkins, Hany Eteiba, Keith Robertson, Colin Berry, Keith G. Oldroyd, Margaret McEntegart

**Affiliations:** ^1^ West of Scotland Heart and Lung Centre Golden Jubilee National Hospital Clydebank UK; ^2^ British Heart Foundation Glasgow Cardiovascular Research Centre Institute of Cardiovascular and Medical Sciences University of Glasgow Glasgow UK; ^3^ Faculty of Medicine University of Newcastle Callaghan NSW Australia

**Keywords:** coronary artery perforation, coronary stent occlusion, covered coronary stent, outcomes, stent thrombosis, Percutaneous Coronary Intervention, Revascularization, Stent

## Abstract

**Background:**

The objective of the GNOCCI (Glasgow Natural History Study of Covered Stent Coronary Interventions) Study was to report the incidence and outcomes of coronary artery perforations over an 18‐year period at a single, high‐volume percutaneous coronary intervention center. We considered both the temporal trends and long‐term outcomes of covered stent deployment.

**Methods and Results:**

We evaluated procedural and long‐term clinical outcomes following coronary perforation in a cohort of 43,343 consecutive percutaneous coronary intervention procedures. Procedural major adverse cardiac events were defined as a composite of death, myocardial infarction, stroke, target vessel revascularization, or cardiac surgery within 24 hours. A total of 161 (0.37%) procedures were complicated by coronary perforation of which 57 (35%) were Ellis grade III. Incidence increased with time over the study period (*r*=0.73; *P*<0.001). Perforation severity was linearly associated with procedural mortality (median 2.9‐year follow‐up): Ellis I (0%), Ellis II (1.7%), Ellis III/IIIB (21%), *P*<0.001. Procedural major adverse cardiac events occurred in 47% of patients with Ellis III/IIIB versus 13.5% of those with Ellis I/II perforations (odds ratio, 5.8; 95% CI, 2.7–12.5; *P*<0.001). Covered stents were associated with an increased risk of stent thrombosis at 2.9‐year follow‐up (Academic Research Consortium definite or probable; 9.1% versus 0.9%; risk ratio, 10.5; 95% CI, 1.1–97; *P*=0.04).

**Conclusions:**

The incidence of coronary perforation increased between 2001 and 2019. Severe perforation was associated with higher procedural major adverse cardiac events and was an independent predictor of long‐term mortality. Although covered stents are a potentially lifesaving treatment, the generation of devices used during the study period was limited by their efficacy and high risk of stent thrombosis.

**Registration Information:**

Clinicaltrials.gov. Identifier: NCT03862352.

**Nonstandard Abbreviations and Acronyms**
CTO: chronic total occlusion
MACE: major adverse cardiac events

**Clinical Perspective**

**What Is New?**

- We report an increasing incidence of coronary perforations over time. Likely reflecting the growth of more complex, high‐risk percutaneous coronary intervention percutaneous coronary intervention in the aging population, together with a significant increase in chronic total occlusion percutaneous coronary intervention and percutaneous coronary intervention requiring calcium modification techniques.
- During long‐term follow‐up, we found that mortality after Ellis grade III coronary perforations was 3 times higher than in patients suffering less severe perforations (adjusted hazard ratio, 3.08; 95% CI, 1.78–5.36; *P*<0.001).
- Although covered stents are a potentially lifesaving treatment, long‐term follow‐up of the previous generation of devices indicates they may be associated with up to a 10‐fold higher risk of stent thrombosis.

**What Are the Clinical Implications?**

- Our observation that severe perforations are associated with 3‐fold higher mortality highlights this group as an at‐risk population that may benefit from more careful follow‐up.
- This study highlights the need to improve outcomes associated with covered stents and the need for appraisal of newer‐generation devices to assess their impact on procedural and long‐term outcomes.

Coronary perforation is a rare but potentially life‐threatening complication of percutaneous coronary intervention (PCI).^1^ It is characterized by iatrogenic injury and resultant rupture of the coronary arterial wall, causing blood to either accumulate outside the vessel (frequently within the pericardial space) or to drain into an adjacent cardiac chamber.^2^

Coronary perforations are typically categorized by the Ellis classification (Figure 1).^3^ Nationwide prospective registry data suggest a relatively low overall incidence of 0.33% (1762/525 359) for PCI performed in the United Kingdom between 2006 and 2013.^4^ However, risk of coronary perforation is higher in calcified and tortuous vessels.^5^ Additionally, there is up to a 10‐fold increase in the frequency of coronary perforations during chronic total occlusion (CTO) PCI, between 2.9 and 4.8% of CTO cases.^6^, ^7^, ^8^

**Figure 1:**
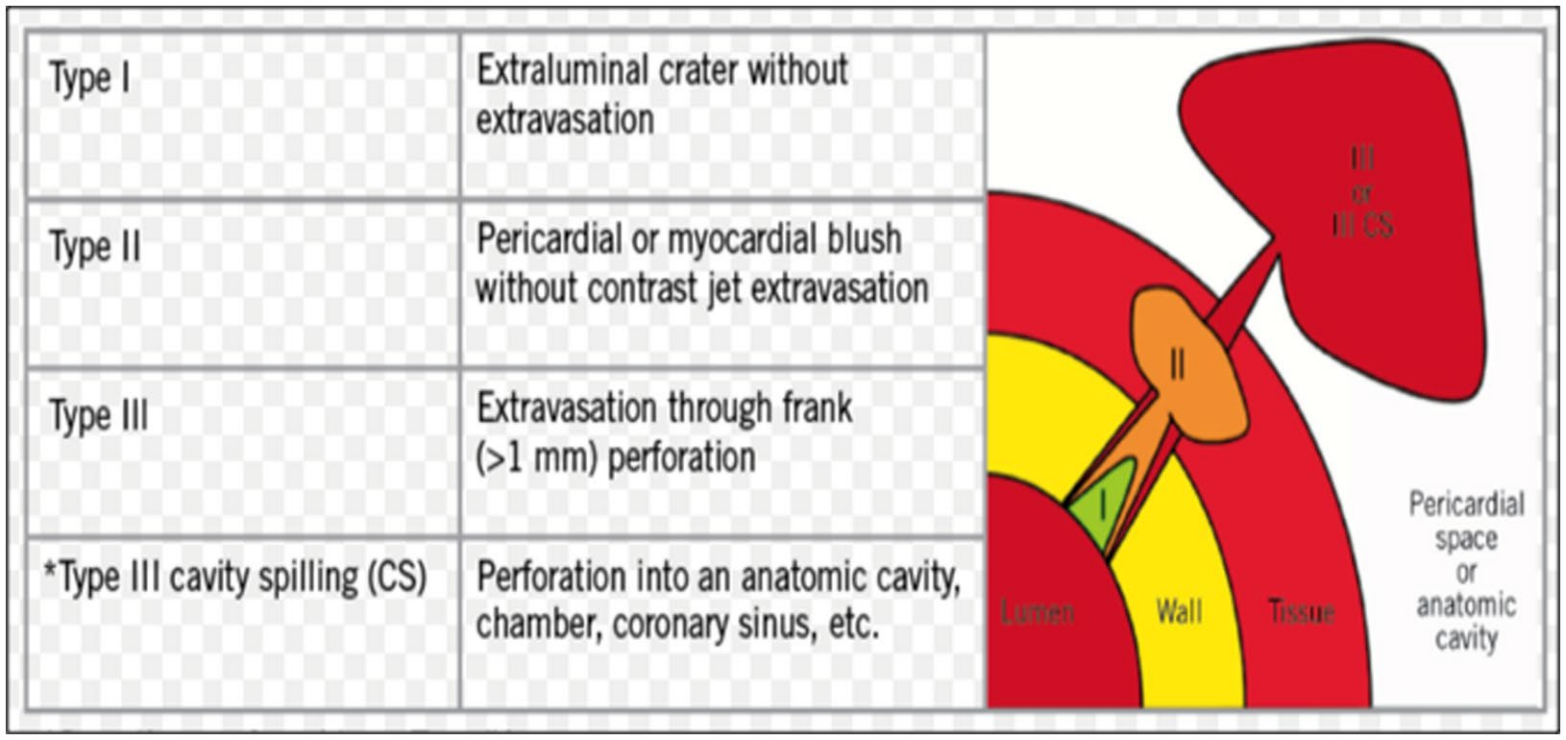
Ellis classification of coronary perforations.

Invasive management is algorithmic and guided by expert consensus.^9^, ^10^ Treatment involves achieving hemostasis at the perforation site with intracoronary balloon tamponade and consideration of a covered coronary stent for Ellis III perforations. Covered stents can be lifesaving but lack data on longer term rates of stent thrombosis and target lesion revascularization and may have reduced durability and safety.^11^ The objective of this study was to report temporal trends in the incidence and long‐term outcomes of coronary artery perforation in a large, consecutive cohort of patients over an 18‐year period at our institution.

## Methods

### Transparency and Openness Promotion

In order to minimize the possibility of unintentionally sharing information that can be used to re‐identify private information, the data that support the findings of this study are available from the corresponding author upon reasonable request rather than a public repository.

### Study Population

We retrospectively analyzed a cohort of 43 343 consecutive PCI procedures performed over 18 years (March 2001 and April 2019) at a single, high‐volume PCI center serving a population of approximately 2 million in the west of Scotland (Clinicaltrials.gov NCT03862352). Regulatory approval for this project was obtained from the local National Health Service trust clinical governance and ethics committee. In view of the high‐volume, retrospective, anonymized design of the study, individual patient consent was not required. This study complies with the Declaration of Helsinki and has full local approval for using subject data.

### Patient Identification

All PCIs complicated by coronary perforation of any severity were identified from an electronic database. The coronary angiogram for each case was then reviewed by 2 physicians (A.J.M. and C.A.), grading the severity of the coronary perforation and gathering information regarding vessel and procedural characteristics. All of the angiograms were then independently reviewed by an interventional cardiologist (T.J.F.), with any conflict being resolved by a further review and consensus involving a senior interventional cardiologist where necessary (M.M.E.).

### Variables

Baseline characteristics and long‐term follow‐up data (median follow up of 2.9 years) were obtained through review and analysis of the local clinical electronic health database by 2 physicians (A.J.M. and C.A.), with historical paper records being accessed when required.

Diabetes was diagnosed according to World Health Organization criteria and included both type 1 and type 2.^12^ Renal impairment was defined as current renal replacement therapy or a serum creatinine >200 μmoL/L. Left ventricular ejection fraction was stratified into 3 categories—good ( > 50%), moderately impaired (30–50%), and severely impaired ( < 30%). Hypertension was defined according to World Health Organization criteria (systolic blood pressure ≥140 mm Hg and/or diastolic blood pressure ≥90 mm Hg) and included patients controlled on antihypertensive therapy.^13^

Coronary lesion complexity was classified according to the American College of Cardiology and American Heart Association lesion classification.^14^ Coronary lesion calcification was classified qualitatively according to the angiographically derived Mintz criteria—with moderate or severe calcification being described as significant.^15^ Coronary perforation severity was categorized by the Ellis classification (Figure 1).^3^ Bleeding outcomes were adjudicated using the Bleeding Academic Research Consortium standardized definitions for cardiovascular clinical trials.^16^

Cardiac tamponade was defined as the presence of a pericardial effusion on echocardiography with either clinical (eg, tachycardia, hypotension, rising jugular venous pressure, electrical alternans, pulsus paradoxus, or muffled heart sounds) or echocardiographic (eg, early diastolic collapse of the right ventricle, late diastolic collapse of the right atrium, abnormal ventricular septal motion, exaggerated respiratory variability in mitral inflow velocity, or swinging of the heart) features of cardiac tamponade.^17^

Procedural major adverse cardiac events (MACE) were defined as a composite of death, myocardial infarction, stroke, target vessel revascularization, or cardiac surgery within 24 hours of the index procedure. Stent thrombosis (definite or probable) was independently determined by 2 cardiologists according to Academic Research Consortium criteria.

### Statistical Analysis

Baseline demographics comparing patients with Ellis I/II and Ellis III grade perforations were compared using Student’s *t*‐test or chi‐square test as appropriate. This was a case‐control evaluation (Ellis III v Ellis I/II as control). Aspects of the procedure associated with the perforation, for example, indication of angiography, type of access, and use of rotablation or intravascular ultrasound between Ellis I/II and Ellis III/IIIB grade perforations were compared between groups using a chi‐square test. Comparisons were also made between groups regarding management of the perforation, for example, use of balloon tamponade and covered stents using a chi‐square test.

We used logistic regression to explore the relationship between severe coronary perforations and procedural MACE, then repeated this adjusting for a limited number of variables prespecified from the literature and determined by consensus that were felt to modify risk of procedural MACE (age, sex, indication, access, diabetes, renal impairment).^18^, ^19^ A Cochran‐Armitage test was used to examine for trend in occurrence of procedural mortality by Ellis classification. Temporal change was evaluated using simple linear regression incorporating year of perforation captured as an ordinal independent variable versus annualized perforation (%) as the dependent variable.

Survival analysis for all‐cause mortality was performed using Cox regression adjusting for baseline variables, looking at predictors of all‐cause mortality at long‐term follow‐up. Covered stents were not included in this model because of colinear association with Ellis III perforation. Proportional hazards assumption was tested by visual inspection of log‐log plots and by test of Schoenfeld residuals and was not violated.

Finally, we performed a prespecified analysis of stent thrombosis in coronary perforation survivors who received a covered stent comparing this to coronary perforation survivors who did not receive a covered stent, using logistic regression to compare odds of acute stent thrombosis.

For all tests, a *P*≤0.05 was considered statistically significant. Statistical analyses were performed using SPSS, Version 26 (IBM Corp., Armonk, NY, USA) and Stata version 16 (Stata Corp., College Station, TX, USA).

## Results

### Patient Characteristics

**Figure 2:**
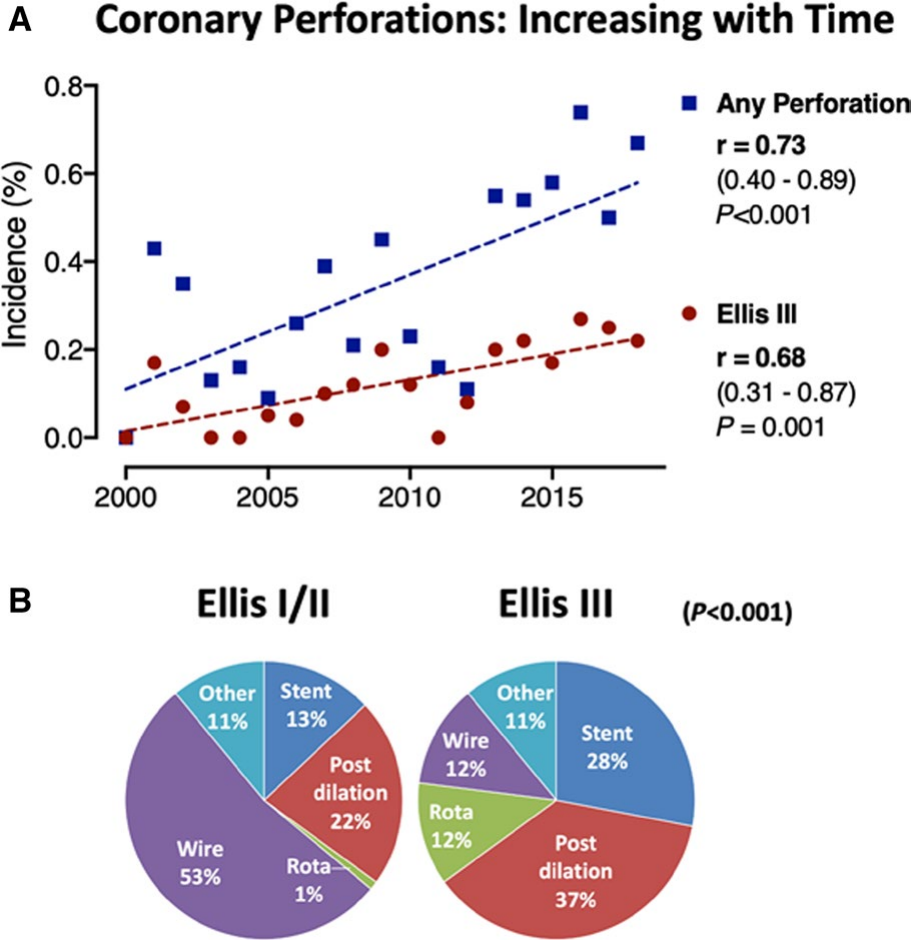
Temporal trend shows increasing incidence of coronary perforation incidence (**A**) and etiology of coronary perforation varies according to severity (**B**). Spearman’s rho demonstrates correlation of incidence with time.

The incidence of coronary perforation was 0.37% (161/43 343 invasive coronary procedures). There was a notable increase in coronary perforation incidence over the 18‐year study period (Figure 2A; *P*<0.001). The mean age of study patients was 69 (SD 11 years) with median follow‐up of 2.9 years.

Patient characteristics stratified by perforation grade (Ellis I/II versus III/IIIB) are described in Table 1. Ellis I/II coronary perforations were more common than type III/IIIB (104 [65%] versus 57 [35%]). Those with Ellis III/IIIB perforations were older (71 [SD 10] versus 67 [SD 11] years). The groups were otherwise similar in terms of comorbidities.

**Table 1:** Patient Demographics

	Ellis I/II	Ellis III/IIIB	*P* value
Number	104	57	
Demographics			
Female sex, %	43 (41.3)	24 (42.1)	0.93
Age, y	67 ± 11	71 ± 10	0.027
Body mass index, kg/m^2^	28.6 ± 7.4	26.6 ± 4.0	0.063
Hypertension, %	65 (62.5)	33 (57.9)	0.57
Diabetes, %	15 (14.4)	12 (21.1)	0.28
CKD (%)			
None	100 (96.2)	53 (93.0)	0.68
CKD (creatinine >200)	2 (1.9)	2 (3.5)	
Dialysis	2 (1.9)	2 (3.5)	
Canadian Cardiovascular Society score for angina, %			
1	5 (4.8)	3 (5.3)	0.69
2	18 (17.3)	6 (10.5)	
3	33 (31.7)	18 (31.6)	
4	48 (46.2)	30 (52.6)	
New York Heart Association class, %			
1	57 (54.8)	36 (63.2)	0.44
2	27 (26.0)	12 (21.1)	
3	12 (11.5)	3 (5.3)	
4	8 (7.7)	6 (10.5)	
Left ventricle function, %			
Unknown	15 (14.4)	7 (12.3)	0.88
EF >50%	52 (50.0)	26 (45.6)	
EF 30–49%	29 (27.9)	19 (33.3)	
EF <30%	8 (7.7)	5 (8.8)	
Previous percutaneous coronary intervention, %	24 (23.1)	15 (26.3)	0.65
Previous coronary artery bypass grafting, %	13 (12.5)	9 (15.8)	0.56

Plus‐minus values are means±SD. Percentages may not total 100 due to rounding. CKD indicates chronic kidney disease; and EF, ejection fraction.

### Procedure Details

Table 2 highlights procedural characteristics according to perforation grade. Perforation etiology differed between groups as anticipated (Figure 2B). Most Ellis I/II perforations were wire perforations (53%), compared with 12.3% of Ellis III/IIIB. The most frequent causes of Ellis III/IIIB perforations were balloon postdilation (37%) or stent deployment (28%; Figure 2B).

**Table 2:** Procedural Considerations

	Ellis I/II	Ellis III/IIIB	*P* value
	(n=104)	(n=57)	
Indication, %			
Stable	51 (49.0)	24 (42.1)	0.61
Acute coronary syndrome	35 (33.7)	20 (35.1)	
ST‐segment–elevation myocardial infarction	18 (17.3)	13 (22.8)	
Arterial access, %			
Femoral	20 (19.2)	10 (17.5)	0.002
Radial	82 (78.8)	38 (66.7)	
Other	1 (1.0)	0 (0.0)	
Radial and femoral	1 (1.0)	9 (15.8)	
Extent coronary artery disease, %			
Single VD	22 (21.2)	8 (14.0)	0.24
Two VD	36 (34.6)	16 (28.1)	
Three VD	46 (44.2)	33 (57.9)	
Intra‐aortic balloon pump, %	3 (2.9)	4 (7.0)	0.22
Temporary pacing line, %	1 (1.0)	2 (3.5)	0.25
Intravascular ultrasound, %	11 (10.6)	12 (21.1)	0.069
Rotablation, %	6 (5.8)	16 (28.1)	<0.001
Perforated vessel, %			
Left main stem	1 (1.0)	2 (3.5)	0.15
Left anterior descending	40 (38.5)	32 (56.1)	
Circumflex	17 (16.3)	6 (10.5)	
Right coronary artery	35 (33.7)	10 (17.5)	
Graft	4 (3.8)	3 (5.3)	
Diagonal	1 (1.0)	0 (0.0)	
Obtuse marginal	2 (1.9)	3 (5.3)	
Septal	4 (3.8)	1 (1.8)	
Etiology, %			
Predilatation	11 (10.6%)	5 (8.8%)	<0.001
Stent	13 (12.5%)	16 (28.1%)	
Post‐dilatation	23 (22.1%)	21 (36.8%)	
Rotablation	1 (1.0%)	7 (12.3%)	
Wire	55 (52.9%)	7 (12.3%)	
Other	1 (1.0%)	1 (1.8%)	
Chronic total occlusion, %	20 (19.2)	11 (19.3)	0.99
Significant calcification, %	54 (51.9)	38 (66.7)	0.071

VD indicates vessel disease.

Both Ellis perforation groups had similar proportions of CTO lesions (19.2% Ellis I/II and 19.3% Ellis III/IIIB) and similar prevalence of significant calcification. CTO procedures requiring both femoral and radial arterial access were more likely to be associated with Ellis III/IIIB perforations. Rotational atherectomy was more commonly used during procedures complicated by Ellis III/IIIB compared with Ellis I/II perforations (28.1% v 5.8% [Table 2]).

### Perforation Management

Most patients received an initial trial of balloon tamponade or conservative management in both Ellis I/II and Ellis III/IIIB perforations (76% versus 86%, respectively) (Table 3). The use of a covered stent was much more common in Ellis III/IIIB perforations than in Ellis I/II (63.2% versus 2.9%). Covered stent insertion was attempted in 45 patients, deployed in 39, and successfully sealed the perforation in 30 (67%) patients. When a covered stent was deployed it achieved hemostasis in 75% (27/36) of Ellis III/IIIB perforations. Unsuccessful covered stent deployment (N=6) was either due to an inability to track the stent to the site of perforation or device/guide catheter incompatibility. These were all earlier generation covered stents devices—Graftmaster (Abbott, Redwood City, CA, USA) and Aneugraft (ITGI Medical, Nir Akiva, Israel). Patients with unsuccessful covered stent deployment or continued bleeding after successful insertion (n=15/45), had a very high mortality within 24 hours of the procedure (n=7/15; 47%). The use of other interventional management options including coil or fat embolization and heparin reversal were similar between groups. Patients with Ellis III/IIIB perforations were much more likely to require surgical management (14% versus 1.9%; *P*=0.002).

**Table 3:** Management of Perforation

Management of perforation	Ellis I/II	Ellis III/IIIB	*P* value
Balloon tamponade/conservative treatment, %	79 (76.0)	49 (86.0)	0.13
Covered stent inserted, %	3 (2.9)	36 (63.2)	<0.001
Covered stent successful hemostasis	100%	75%	
Coil, %	0 (0.0)	1 (1.8)	0.18
Fat embolization, %	3 (2.9)	1 (1.8)	0.66
Cardiac surgery, %	2 (1.9)	8 (14.0)	0.002
Multiple treatments required, %	8 (7.7)	30 (52.6)	<0.001
Heparin reversed, %	17 (16.3)	10 (17.5)	0.85

*P* value from Pearson’s chi square test for difference between groups.

### In‐Hospital Complications Following Coronary Perforation

Ellis III/IIIB perforations were associated with very high rates of procedural MACE compared with Ellis I/II (47% versus 14%; odds ratio [OR], 5.8; 95% CI, 2.7–12.5; *P*<0.001). This remained significant after adjustment of between group differences (Table 4).

**Table 4:** In‐Hospital Complications

	Ellis I/II	Ellis III	*P* value^*^
Procedural major adverse cardiac events^†^	14 (13.5%)	27 (47.4%)	<0.001
Odds ratio (Ellis III vs I/II ) (95% CI)	5.8 (2.7–12.5)		
Adjusted odds ratio^‡^ (Ellis III vs I/II) (95% CI)	8.4 (3.5–20.5)		
Cardiac surgery	2 (1.9%)	8 (14.0%)	0.002
Death within 24 hours	1 (1.0%)	12 (21.1%)	<0.001
Stroke	1 (1.0%)	0 (0.0%)	0.46
Reintervention	5 (4.8%)	6 (10.5%)	0.17
Myocardial infarction	7 (6.7%)	13 (22.8%)	0.003
Major vessel occlusion	3 (2.9%)	12 (21.1%)	<0.001
Shock/intra‐aortic balloon pump insertion	8 (7.7%)	28 (49.1%)	<0.001
Tamponade			
No	92 (88.5%)	32 (56.1%)	<0.001
Acute	4 (3.8%)	23 (40.4%)	
Delayed	8 (7.7%)	2 (3.5%)	
Acute renal failure	2 (1.9%)	8 (14.0%)	0.002

* *P* value given for chi‐square test of difference between groups.

^†^ Defined as a composite of death, myocardial infarction, stroke, target vessel revascularization, or cardiac surgery within 24 hours)

^‡^ Adjusted for age, sex, indication for percutaneous coronary intervention, type of arterial access, diabetes, chronic kidney disease.

Perforation severity was linearly associated with procedural mortality: Ellis I (0%), Ellis II (1.7%), Ellis III/IIIB (21%), *P*<0.001. All components of procedural MACE were more common in patients with Ellis III/IIIB perforations, except for stroke, which occurred in only 1 patient. In‐hospital acute renal failure and cardiac tamponade were both more common in Ellis III/IIIB than Ellis I/II perforations (renal failure 14.0% versus 1.9%; tamponade 40.4% versus 3.8%; *P*<0.001; Table 5).

**Table 5:** Hazard Ratio for Occurrence of Death

		Hazard ratio	95% CI		*P* value
Ellis III/IIIB		3.08	1.78	5.36	<0.001
Age (per 10‐year increment)		1.92	1.42	2.59	<0.001
Female sex		0.53	0.30	0.94	0.03
Indication					
	Stable	REF			
	Acute coronary syndrome	1.89	1.01	3.55	0.05
	Emergency—ST‐segment–elevation myocardial infarction or cardiogenic shock	2.6	1.26	5.36	0.01
Arterial access					
	Radial	REF			
	Femoral	1.31	0.68	2.53	0.423
	Other/combination	0.41	0.10	1.75	0.228
Diabetes		1.66	0.79	3.48	0.18
Renal impairment					
	None	REF			
	Chronic kidney disease (creatinine >200)	0.95	0.22	4.19	0.95
	Dialysis	1.19	0.28	5.11	0.82

REF indicates reference.

### Long‐Term Outcome Following Coronary Perforation

More than 1 in 5 patients suffered a fatal outcome within 24 hours of a severe coronary perforations (Ellis III versus Ellis I/II, 21% versus 1%; *P*<0.001). The median survival of patients following Ellis III coronary perforation was 4.6 (95% CI, 0.9–13.2) years.

**Figure 3:**
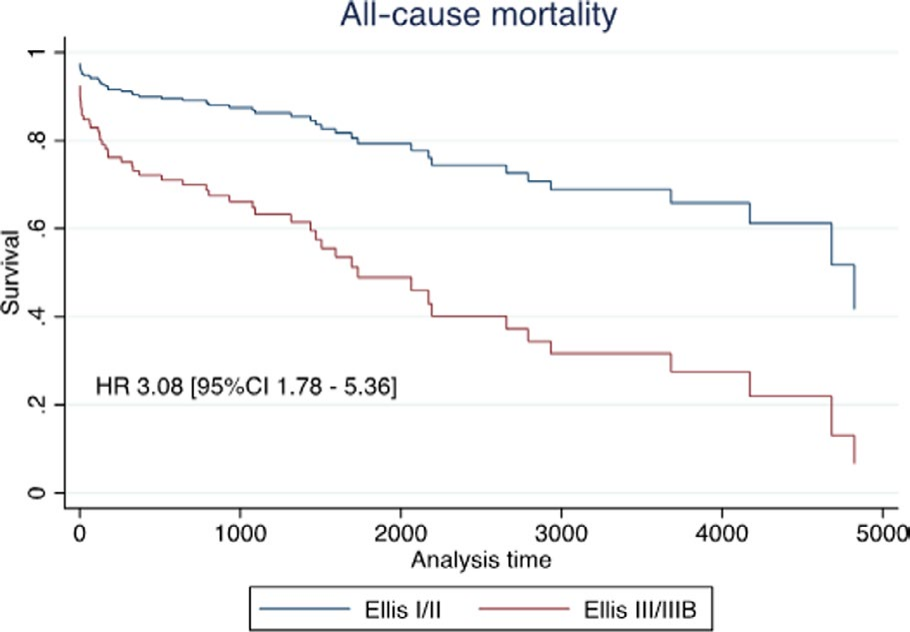
Perforation severity predicts long‐term outcomes. COX regression all‐cause mortality for Ellis I/II and Ellis III/IIIb coronary perforations. Survival curves and hazard ratio from Cox regression analysis for all‐cause mortality including adjustment for age, sex, indication for angiography, type of arterial access, presence of diabetes, and chronic kidney disease. HR indicates hazard ratio.

In multivariable Cox regression analysis severe perforation (Ellis III) was independently associated with all‐cause mortality (HR, 3.08; 95 % CI, 1.78–5.36; *P*<0.001). Other factors associated with long‐term survival following coronary perforation were age (hazard ratio [HR] 1.92 per 10 years, 95% CI, 1.42–2.59; *P*<0.001) and procedural urgency. Emergency procedures (HR, 2.6; 95 % CI, 1.3–5.4; *P*=0.01) and acute coronary syndrome procedures (HR, 1.9; 95 % CI, 1.0–3.6; *P*=0.05) were associated with reduced long‐term survival compared with stable patients. Survival stratified by grade of coronary artery perforation is illustrated in Figure 3.

### Long‐Term Outcome Following Covered Coronary Stent

The incidence of any stent thrombosis (Academic Research Consortium definite or probable) in patients who survived the first 24 hours following covered stent insertion was 9% at median follow‐up 2.9 years. Patients who received a covered stent had an increased risk of acute stent thrombosis compared with those managed without a covered stent (Academic Research Consortium definite or probable; 9.1% versus 0.9%; OR, 10.1; 95% CI, 1.02–99.93; *P*=0.048).

## Discussion

In this analysis we identified an increasing incidence of coronary perforations at our institution, from approximately 0.2% in 2000 to over 0.6% in recent years. Second, we highlight the prognostic utility of the Ellis classification system in determining both short‐term in‐hospital outcomes and long‐term survival. Specifically, we observed that over 20% of patients with Ellis III perforations died within 24 hours, and almost 50% of major perforations were associated with procedural MACE. In addition, we contribute to the evidence base highlighting the challenges and potential hazards associated with earlier generation covered stents.

### Increasing Incidence of Coronary Perforation

Our study revealed an overall incidence of coronary perforation of 0.37% consistent with nationwide UK data (0.33%) and estimated incidence from pooled studies (0.43%).^4^, ^20^ We observed a trend of increasing coronary perforation over time, from an incidence of approximately 0.2% to 0.6%, likely reflecting the growth of more complex, high‐risk PCI in the aging population, together with a significant increase in CTO PCI and PCI requiring calcium modification techniques.^21^, ^22^

During the first‐generation drug‐eluting stent era, coronary perforations were reducing in frequency.^23^ Some more recent reports^24^, ^25^ have shown either static incidence or did not specifically analyze trends over time. An analysis from the UK national database (up to 2013) also reported a trend toward an increased incidence of perforations although this was not statistically significant.^3^ Although our data did not allow for analysis of temporal mortality trends after coronary perforation, the UK national data showed a significant interaction between time and mortality whereby perforations were increasingly likely to result in death (mortality varied from 6.6% to 15.5% with a significant upward trend; *P*=0.049). This highlights the clinical importance of this subject and the need for further improvements in the management this potentially fatal complication.

### Ellis III Perforation and Mortality

Perforation severity was linearly associated with procedural mortality (Ellis I, 0%; Ellis II, 1.7%; Ellis III/IIIB, 21%; *P*<0.001]. This substantial procedural mortality after Ellis III perforations (21%) is consistent with the pooled estimated mortality from a historical meta‐analysis of 16 studies (21.3%),^1^ whereas more recent studies have reported in‐hospital mortality rates of between 15% and 44%.^20^, ^22^ Larger cohort studies that do not classify severity report an overall in‐hospital mortality of around 8% for all coronary perforation.^4^, ^24^

During long‐term follow‐up, we found that mortality after Ellis grade III coronary perforations was 3 times higher than in patients suffering less severe perforations (adjusted HR, 3.08; 95 % CI, 1.78–5.36; *P*<0.001). This is a novel finding and merits further consideration. The precise mechanisms for this are uncertain but relevant factors may include suboptimal revascularization due to procedure interruption secondary to perforation, concomitant complications (eg, vascular complications, periprocedural myocardial infarction, major bleeding), and restenosis or thrombosis of covered stents. Although coronary perforation has previously been reported to have an adverse prognostic impact on long‐term survival (HR, 1.35; *P*<0.001),^26^ our observation that severe perforations are associated with 3‐fold higher mortality highlights this group as an at‐risk population that may benefit from more careful follow‐up.

Our data highlight the challenges and limitations of earlier generation polytetrafluoroethylene‐covered stents, typically constructed with polytetrafluoroethylene sandwiched between 2 stents.^27^ Successful stent deployment and establishment of hemostasis were surprisingly low at 66%, in part because of challenges with delivery and guide catheter size compatibility issues. The recent availability of a 5‐French‐compatible device (PK Papyrus, Biotronik) has been a significant development, which was not available at our center during the studied time period. The design comprises a single stent covered with a nonwoven, electrospun polyurethane material that creates a thin and highly elastic membrane, resulting in the device being lower profile and easier to deliver.^24^ Nevertheless, more data on its efficacy following severe coronary perforation and long‐term safety in comparison to polytetrafluoroethylene devices are required.

### Covered Stent Thrombosis

The incidence of covered stent thrombosis at almost 3 years follow‐up was high (Academic Research Consortium definite or probable; 9.1% versus 0.9% in patients without covered stent insertion; relative risk, 10.5; *P*=0.04). There are very limited data in the existing literature, with previously reported stent thrombosis rates between 3.4 and 4% at 1 year and 8.6% at 3 years.^25^, ^27^ This highlights the need to improve outcomes associated with covered stents, possibly through increased use of intravascular imaging, longer duration of dual antiplatelet therapy, and the availability of newer‐generation devices.

## Conclusions

In this single‐center study we report an increasing incidence of coronary perforations over time. Perforations were associated with high procedural mortality and among survivors a significant risk of long‐term MACE. Severe coronary perforations (Ellis III) were associated with a 21% risk of procedural death and among survivors had a legacy impact on long‐term survival. Covered stents are a potentially lifesaving treatment, but long‐term follow‐up of the previous generation of devices indicates they may be associated with up to a 10‐fold higher risk of stent thrombosis. Further research with newer generation devices is required to assess their impact on procedural and long‐term outcomes.

### Limitations

The scope of this study is limited by its retrospective and single‐center design. The impact of these design limitations has been minimized by robust adjudication of both clinical and procedural data by experienced researchers, although accuracy is reliant on recording of routine data and self‐reported adverse events may vary between operators and is a potential source of bias. No statistical correction for multiple testing was undertaken, increasing the risk of type I error.

## Sources of Funding

T.J.F. and C.A. have research funding from the British Heart Foundation (RE/18/6134217). C.B. and A.J.M. have research funding from the Medical Research Council (MR/S018905/1).

## Disclosures

C.B. is employed by the University of Glasgow, which holds consultancy and research agreements with companies that have commercial interests in the diagnosis and treatment of angina. The companies include Abbott Vascular, AstraZeneca, Boehringer Ingelheim, HeartFlow, Medyria, Menarini Pharmaceuticals, Novartis, and Siemens Healthcare. K.G.O. has received consultant and speaker fees from Abbott Vascular and Volcano Corporation. S.W. has received consultant and speaker fees from Boston Scientific. M.M. has received consultant and speaker fees from Boston Scientific and Abbott Vascular. None of these companies has had any involvement with this study. The remaining authors have no disclosures to report.
